# Gradient-Based Time-Extended Potential Field Method for Real-Time Path Planning in Infrastructure-Based Cooperative Driving Systems

**DOI:** 10.3390/s25175601

**Published:** 2025-09-08

**Authors:** Jakyung Ko, Inchul Yang

**Affiliations:** 1 Department of Civil and Environmental Engineering, University of Science and Technology, Daejeon 34113, Republic of Korea; jakyungko@kict.re.kr; 2 Department of Highway and Transportation, Korea Institute of Civil Engineering and Building Technology, Goyang-si 10223, Republic of Korea

**Keywords:** automated driving, path planning, potential field, cooperative automated driving, infrastructure-based cooperative driving

## Abstract

This study proposes a real-time path generation method called the Gradient-based Time-extended Potential Field (GT-PF) for cooperative autonomous driving environments. The proposed approach models the road environment and dynamic obstacles as a time-variant potential field and generates safe and feasible paths by tracing the negative gradient of the field, which corresponds to the direction of steepest descent. In contrast to conventional sampling-based or optimization-based methods, the proposed PF framework enables lightweight computation and continuous trajectory generation in spatiotemporal domains. Furthermore, a velocity-oriented bias is introduced in the PF formulation to ensure that the generated paths satisfy the vehicle’s kinematic constraints and desired cruising behavior. The effectiveness of the proposed method is verified through comparative simulations against a sampling-based Rapidly exploring Random Tree (RRT) planner. Results demonstrate that the GT-PF approach exhibits superior performance in terms of runtime efficiency and safety. The system is particularly suitable for RSU (Roadside Unit)-based infrastructure control in real-time traffic environments. Future work includes the extension to complex urban scenarios, integration with multi-agent planning frameworks, and deployment in sensor-fused cooperative perception systems.

## 1. Introduction

With the development of autonomous vehicles and cooperative driving technologies based on information sharing between vehicles and infrastructure (V2I), the need for real-time generation of feasible, fine-grained paths on road networks has grown significantly [1,2]. In high-level cooperative driving, vehicles not only exchange environmental and dynamic information but also share driving intentions to negotiate and derive mutually understandable trajectories [3].

These trajectories must go beyond simple goal-reaching; they should reflect the dynamic behavior of preceding vehicles, road geometry, and various driving intentions such as lane changes or overtaking. Therefore, a local path must ensure both dynamic feasibility and safety while remaining lightweight enough to be processed by infrastructure-side devices like roadside units (RSUs) in real-time environments [4].

Several methods have been proposed for local path generation, including sampling-based, optimization-based, trajectory regeneration-based, and potential field (PF)-based approaches [5]. Sampling-based methods such as the Rapidly exploring Random Tree (RRT) and Probabilistic Roadmap Method (PRM) can explore diverse routes through repeated random node generation and connection, but they suffer from exponential increases in computational cost as resolution or search width increases—making them unsuitable for real-time applications [6]. Optimization-based methods, including Model Predictive Control (MPC) and Iterative Linear Quadratic Regulator (iLQR), can produce high-quality paths by considering both cost functions and dynamic constraints. However, as the complexity of vehicle models or constraints increases, the computational burden becomes significant, posing challenges for real-time deployment on edge devices or infrastructure [7]. Trajectory regeneration-based methods, such as the Dynamic Window Approach (DWA), evaluate multiple trajectory candidates based on distance to obstacles and cost functions for linear and angular velocity combinations. Yet, these methods are sensitive to the number of samples and evaluation metrics, which can lead to increased computation time and difficulties in trajectory tracking [8].

Among these, PF-based methods have received attention for their simple structure and high computational efficiency in real-time cooperative driving environments. These methods synthesize attractive forces (towards the goal) and repulsive forces (from obstacles, lane boundaries, or other vehicles) into a spatial potential field. Movement directions are then derived from the gradient of this field, guiding vehicles along paths where the potential value decreases most rapidly. This structure allows for real-time obstacle avoidance and motion decision-making using only gradient calculations, offering high efficiency, even in complex multi-agent environments.

However, traditional PF-based methods lack temporal or velocity considerations and do not explicitly reflect vehicle kinematic constraints. As a result, if path planning is based solely on spatial coordinates (x, y), the derived trajectories may be physically infeasible or may violate constraints related to speed, acceleration, or steering angle.

To address these limitations, recent research has extended PF-based path planning methods to enhance dynamic feasibility. Examples include incorporating time as a third axis (i.e., spatiotemporal fields (x, y, t)), introducing constraints for desired velocity, acceleration, or jerk, and applying penalties to unstable or uncomfortable trajectories while assigning weights to dynamically favorable paths.

Especially in cooperative driving environments, there is a growing need to maintain real-time responsiveness while ensuring safety, comfort, and computational efficiency. Accordingly, such enhancements must be integrated into PF-based frameworks to improve trajectory quality and practical deploy ability.

This study focuses on improving computational efficiency in a 3D PF-based path generation framework that accounts for real-time infrastructure-assisted cooperative driving and dynamic traffic environments. Our proposed method builds a PF in a space that integrates both spatial and temporal axes, thereby capturing the real-time movements of preceding vehicles and changes in road conditions over time.

While some prior studies have implemented 3D PF frameworks, they often rely on sampling-based or MPC-based approaches. Sampling-based searches such as RRT are known to incur high computational costs and exhibit strong dependency on sample count, making them difficult to apply in real-time within high-dimensional spaces.

In contrast, our method, termed the Gradient-based Time-extended Potential Field (GT-PF), introduces a gradient-based path following scheme for potential minimization, combined with a time–distance conversion factor derived from desired speed and a bias toward favorable kinematic states. This structure enables low complexity path generation while incorporating real-time feasibility based on actual vehicle characteristics, such as preferred speed, acceleration capacity, and physical constraints. The proposed GT-PF framework generates dynamically feasible reference trajectories that serve as mission-level guidance rather than low level control commands (e.g., steering or throttle). These trajectories are computed at the infrastructure edge and transmitted via cooperative messages, enabling individual vehicles to execute their own local control strategies while adhering to infrastructure guided paths. By avoiding the computational complexity of direct control optimization, this approach prioritizes real time responsiveness and compatibility with cooperative driving architectures, making it highly suitable for large scale deployment in connected traffic environments.

Through simulation, we compare the proposed GT-PF method against sampling-based planners and analyze performance in terms of real-time capability, safety, and practical deployability. Our findings demonstrate that the GT-PF method can generate executable and stable paths within short computation times, thereby offering a viable solution for infrastructure-supported cooperative driving systems.

## 2. Related Works and Research Trends

### 2.1. Potential Field Applications in the Transportation Domain

**Figure 1:**
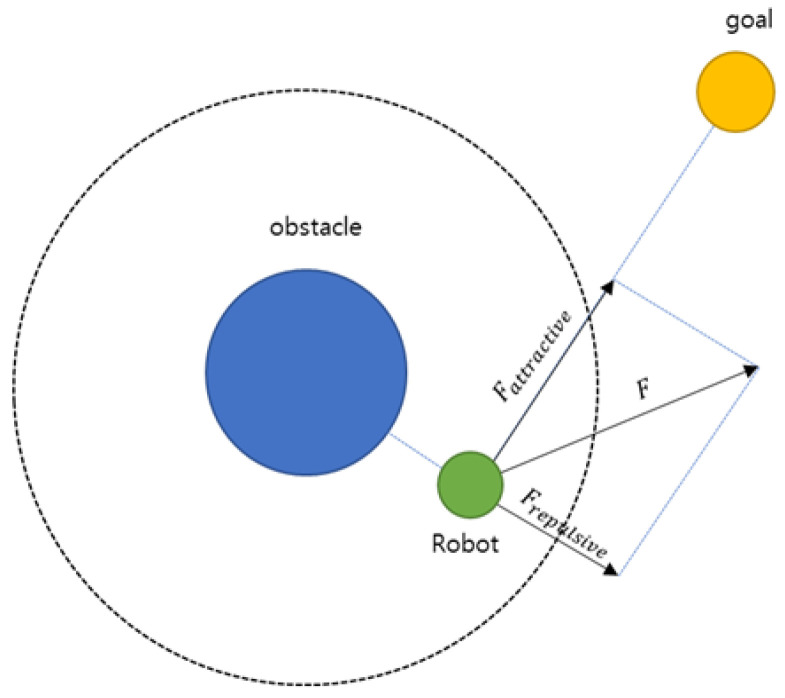
Attractive force and repulsive force in APF [10].

The theory of Potential Fields originates from Khatib’s Artificial Potential Field (APF) model [9]. As illustrated in Figure 1, APF combines attractive forces that guide a vehicle toward its destination and repulsive forces that prevent collisions with obstacles. By synthesizing these two forces, the model simplifies environmental interactions on a two-dimensional plane, enabling path generation even in complex surroundings.

To overcome the limitations of applying APF to flat 2D spaces and to account for the unique characteristics of road environments, numerous studies have proposed extensions to the original model. Several researchers have incorporated road lanes, boundaries, infrastructure, and surrounding vehicles as sources of attractive and repulsive potentials [11,12,13,14,15].

To address interactions with dynamic obstacles such as moving vehicles, the Yukawa potential has been adopted to simulate repulsion based on velocity and acceleration, enabling predictive modeling under complex road scenarios [16]. In addition, driver-perceived risk was modeled to generate potential fields that better reflect real-world safety concerns [17,18]. These risk-based models have been applied not only to path planning but also to driver behavior analysis and safety evaluation [19,20,21,22,23].

In summary, potential field theory has evolved from a method for generating collision-free paths on 2D planes to a modeling framework that can represent real-world environmental factors (e.g., road geometry, pedestrian flow, inter-vehicle interactions). It has recently been applied to collaborative driving, real-time local path planning, and multi-CAV safety assessment systems.

### 2.2. Path Generation Using Potential Fields

A common method for path generation using potential fields involves tracking the direction of the steepest descent in the composite potential field, that is, the direction of the field’s negative gradient. This approach allows various spatial elements such as road boundaries, obstacles, and target points to be simultaneously embedded in the potential field via combinations of attractive and repulsive forces. As a result, it enables the system to select the direction with the lowest risk in real time, even in complex road environments. This method features a lightweight structure and relatively high operational efficiency.

However, the potential field approach also poses limitations in terms of continuity and the applicability of the generated paths. In particular, the presence of surrounding obstacles and road structures can lead to local minima in the potential field, causing the vehicle to become trapped and halting path generation. Furthermore, because path generation is typically conducted based on instantaneous calculations, potential field methods struggle to reflect environmental changes over time, and it is difficult to guarantee the kinematic feasibility of the resulting path.

To overcome these shortcomings, various attempts have been made to hybridize the potential field approach with other path planning techniques. Some studies have introduced potential field–based cost terms into A* search algorithms to increase costs near obstacles and enforce avoidance [24,25]. Others proposed hybrid structures that combine APF-based A* search with Bézier curve smoothing to improve obstacle avoidance for both vehicles and pedestrians [26]. In the context of sampling-based algorithms such as RRT and RRT*, the potential field has been utilized to provide attractive bias toward the goal and repulsive bias from obstacles, addressing inefficiencies such as random expansion and oscillatory behavior [27,28,29].

To enhance real-time applicability and feasibility, several studies have integrated potential fields with optimal control frameworks. Some works have proposed combining Model Predictive Control (MPC) with potential field–based path planning to optimize trajectory, speed, and steering simultaneously, using cost functions that incorporate potential gradients, tracking errors, and dynamic constraints [30]. Various implementations of such hybrid methods have been demonstrated for two- and four-wheel vehicle models, real-time obstacle avoidance, and cooperative driving across complex road geometries [31,32,33,34,35,36,37].

Beyond path generation, velocity planning methods based on space–time (S-T) graphs have been introduced to assign realistic speed profiles while avoiding dynamic obstacles and optimizing comfort and safety criteria such as acceleration, jerk, and headway [38]. To overcome the limitations of static PFs, recent studies have extended PFs into spatiotemporal domains, combining them with sampling-based planners like RRT to enhance planning performance in dynamically evolving environments [39]. These hybrid approaches demonstrate that combining PF with optimal control, velocity profiling, and spatiotemporal planning can significantly improve the feasibility, safety, and practicality of path planning in real-world autonomous driving contexts.

In summary, while traditional PF methods can rapidly determine driving direction through gradient descent, their limitations regarding continuity and feasibility have led to the development of extended hybrid approaches. These combine PF with path search or control-based methods, contributing to improvements in risk-aware, smooth, and feasible path generation. However, many of these methods still require high computational effort, limiting their real-time applicability in infrastructure-based or edge-device systems. Moreover, they do not fundamentally resolve the gap between PF space and time-domain constraints. As a result, there remains a need for a PF-based path generation approach that can satisfy the demands of real-time computation, execution feasibility, and deployability in cooperative autonomous driving environments.

GT-PF inherits several conceptual ideas from prior works—such as predictive extensions and risk-sensitive field design—but reorients them toward a lightweight, infrastructure-centric framework. Unlike approaches focused on low-level control integration, GT-PF prioritizes rapid reference trajectory generation at the infrastructure edge while embedding dynamic feasibility directly into the field formulation rather than through subsequent optimization. This design choice enables compatibility with real time cooperative driving environments and distinguishes GT-PF from previous spatiotemporal PF methods.

To clarify the distinction between GT-PF and prior spatiotemporal PF approaches, Table 1 summarizes key methodological differences. Unlike [30,36], which ensure feasibility through optimization-based controllers, GT-PF embeds speed and acceleration considerations directly into the potential function, eliminating the need for complex control integration. Compared to [39], which relies on computationally expensive sampling-based search, GT-PF employs gradient driven exploration with speed scaled time normalization, reducing computational overhead while maintaining spatiotemporal continuity. These features enable real-time trajectory generation on infrastructure edge devices, ensuring compatibility with cooperative driving environments.

## 3. Proposed Method

### 3.1. Overview

This chapter outlines the proposed path generation method, called the Gradient-based Time-extended Potential Field (GT-PF), which is designed to ensure both feasibility and safety in real-time infrastructure-assisted cooperative driving environments. The GT-PF method constructs a time-extended spatiotemporal potential field that captures dynamic environmental risks and vehicle preferences, while maintaining low computational complexity.

First, we describe how various road and traffic elements—such as lane boundaries, fixed structures, and dynamic vehicles—are encoded into the potential field using attractive or repulsive potential functions. These forces are superimposed to form a composite field that represents the overall navigational cost across the drivable area.

Next, we explain how the temporal dimension is integrated into spatial coordinates to construct a spatiotemporal potential field in (x, y, t) space. This time-extended formulation allows the field to reflect the evolution of traffic and risk over time, enabling predictive path generation.

Finally, we describe how path generation is carried out on the constructed time-extended spatiotemporal field by tracing the gradient (steepest descent) of the field, which points in the direction of the greatest reduction in potential. To increase the practicality of the generated path, we introduce an additional mechanism that reflects the vehicle’s desired kinematic states (such as target velocity), thereby guiding the path planning toward dynamically feasible and smooth trajectories.

The overall process consists of

- Constructing composite potential functions from environmental elements;
- Integrating time into spatial potential field to form a time-extended spatiotemporal field;
- Generating a time-parameterized trajectory by following the negative gradient with added kinematic bias.

Through this structure, the GT-PF method achieves efficient computation, policy-aware trajectory shaping, and practical stability—even under complex multi-agent traffic dynamics.

### 3.2. Construction of a PF Based on Road Environment

#### 3.2.1. Potential for Surrounding Vehicles

In this study, we construct a vehicle-based potential that reflects both the dynamic characteristics of the vehicle and the structured nature of roads divided by lanes. To this end, we apply the Yukawa potential, which models a decaying repulsive field with distance, while incorporating the vehicle’s velocity and acceleration to asymmetrically represent the front-rear and lateral risk zones around the vehicle [10]. The potential is defined as follows:

(1) $$U_{vehicle}=\frac{k_{v}\cdot v+\tau }{s^{\prime }+\epsilon_{1}}\times \exp\left(\frac{1}{v+\epsilon_{2}}\left(\frac{a\left(x-x_{0}\right)}{\left|a\right|+\epsilon_{3}}-s^{\prime }\right)\right)$$

Here, *s*′ represents the *virtual distance* between a point and the vehicle’s center ($x_{0}$, $y_{0}$) which decreases the potential as the distance increases. $k_{v}$ is a velocity coefficient that scales the potential by vehicle speed v, while τ is a safety distance coefficient determining the influence from a stationary vehicle. Higher vehicle speeds result in a broader and more intense potential distribution. The acceleration a determines the extent of influence in the forward or rearward direction. A small positive constant *ϵ* is included to prevent divergence of the potential at zero distance.

The virtual distance $s^{\prime }$ is calculated by considering both longitudinal and lateral distances between the vehicle and a target point. These are combined as

(2) $$s=\sqrt{{\left(x-x_{0}\right)}^{2}+{\left(y-y_{0}\right)}^{2}}\;s^{\prime }=\sqrt{{c_{1}^{2}(x-x_{0})}^{2}+{c_{2}^{2}(y-y_{0})}^{2}}$$

Since vehicles cannot move laterally as freely as longitudinally, the influence in the lateral direction is downscaled. Based on the findings in [16], a 30 m difference in the longitudinal direction is treated equivalently to a 2 m difference laterally. Accordingly, the parameter $c_{1},\;c_{2}$ is introduced to scale the lateral influence. Accordingly, we apply the parameter $c_{1},\;c_{2}$ to scale the lateral effect.

#### 3.2.2. Potential for Road Environment

1. Lane Marking Potential

The lane potential enforces lane-keeping behavior by applying a virtual repulsive force when vehicles deviate from the center of their designated lane. This force is modeled to be stronger near solid yellow lines (center divider) than near dashed white lines (lane change allowed). In this study, we apply repulsive forces to white lines at y = 3.5 and yellow lines at y = 0 and y = 7.0, as expressed in the following equation:

(3) $$U_{lane}=\sum_{i,j}^{n}A_{i}\exp\left(-\frac{d_{lane}^{2}}{2\sigma^{2}}\right),\;d_{lane}=y_{lane}-y$$

where $y_{lane}$ is the coordinate of each lane line and $A_{i}$ denotes the repulsive strength for each lane, which may vary depending on whether the lane marking is yellow (e.g., centerline or median) or white (e.g., standard lane divider). The parameter σ determines both the effective range and the steepness of the repulsive influence.

2. Road Boundary Potential

The road boundary potential is designed to prevent vehicles from departing from the drivable road area. At the edge of the road, the potential diverges to infinity. We incorporate a buffer zone of 1 m from the edge, following the standard shoulder width in road infrastructure design, and apply the following potential function to the boundary:

(4) $$U_{boundary}=\sum_{j}^{2}\frac{1}{2}\eta {\left(\frac{1}{d_{boundary}}\right)}^{2},\;d_{boundary}=y_{boundary}-y$$

where $y_{boundary}$ denotes the coordinate of the road boundary, and η is a scaling factor that determines the magnitude of the repulsive potential. As the distance to the boundary decreases, the potential rises sharply, thereby generating a strong repulsive force that prevents the vehicle from approaching unsafe edge regions.

#### 3.2.3. Goal Attraction Potential

Assuming the current position of the vehicle is p = (x, y) and the goal is located at $p_{goal}=(x_{goal},y_{goal})$, the attractive potential guiding the vehicle toward the goal is defined as

(5) $$U_{goal}=\frac{1}{2}k_{goal}\cdot {\left\|p-p_{goal}\right\|}^{2}$$

Here, $k_{goal}$ is a constant representing the attractive strength toward the goal. In our implementation, the target point is defined at (x, y) = (300, 5.25), corresponding to the center of the goal lane. The potential ensures gradual return to the center even after lane changes.

**Figure 2:**
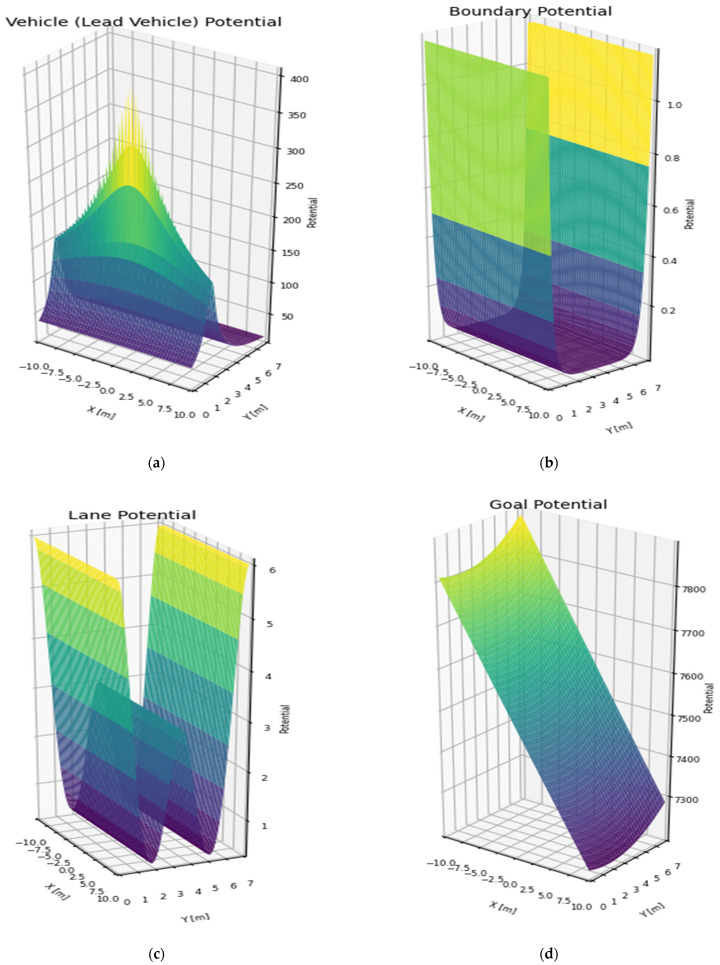
PF: (**a**) lead vehicle; (**b**) road boundaries; (**c**) lane markings; (**d**) goal attraction. Note: The color variations are used only to visually represent changes in intensity and may differ from the actual quantitative values.

Figure 2 illustrates the spatial distribution of each component of the PF:

#### 3.2.4. Composite PF

The final PF used in this study is a weighted summation of the above components and is expressed as

(6) $$U_{total}\left(x,y\right)=w_{1}U_{vehicle}\left(x,y\right)+w_{2}U_{lane}\left(y\right)+w_{3}U_{boundary}\left(y\right)+w_{4}U_{goal}(x,y)$$

Here, $w_{1}$ through $w_{4}$ denote the weights used to scale the relative influence of each potential component.

**Figure 3:**
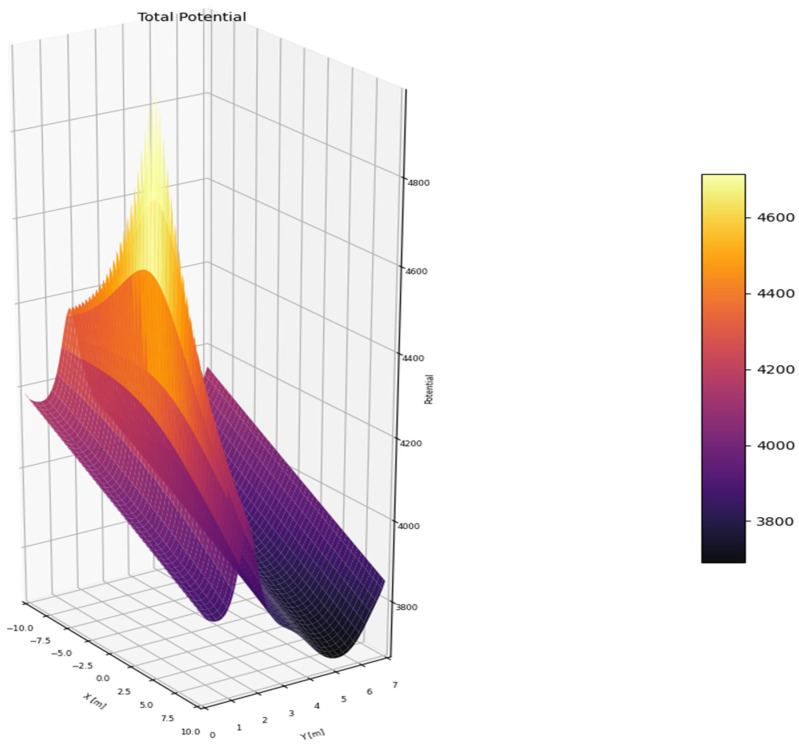
Superposed total PF as the sum of all component fields.

As illustrated in Figure 3, the potential value is minimized at the center of the lane and around the goal location, while high potential values emerge near road boundaries and surrounding vehicles. This design ensures both safety and trajectory stability. The resulting PF is used as the input for the path generation algorithm discussed in the next chapter, contributing to the safe and efficient motion planning of the vehicle.

#### 3.2.5. Rationale and Empirical Validation of Parameter Settings

The parameters employed in the potential function were not obtained through a rigorous optimization process; rather, they were determined within a reasonable range based on theoretical rationale and reference to prior studies. Accordingly, this section focuses on the functional roles of these parameters in path planning and the underlying principles guiding their adjustment.

The vehicle potential was formulated following the structure proposed in previous research, but the parameters were tuned in this study with particular emphasis on path generation and the initiation of avoidance maneuvers. Among these, the combination of τ and $k_{v}$ constitutes a critical factor governing the sensitivity of safety distance characteristics and the initiation threshold for evasive actions. The term τ functions as a base component, operating independently of vehicle speed and determining the fundamental scale of the potential function. In other words, it serves as the reference point for initiating behavior under stationary conditions (v = 0). The parameter $\varepsilon_{2}$, in conjunction with τ, prevents numerical divergence by mitigating denominator shrinkage in the near-field region.

The PF surrounding a stationary obstacle vehicle is expressed as follows:

(7) $$U_{vehicle}=\frac{\tau }{s^{\prime }+\epsilon_{1}}\times \exp\left(\frac{s^{\prime }}{\epsilon_{2}}\right)$$

**Figure 4:**
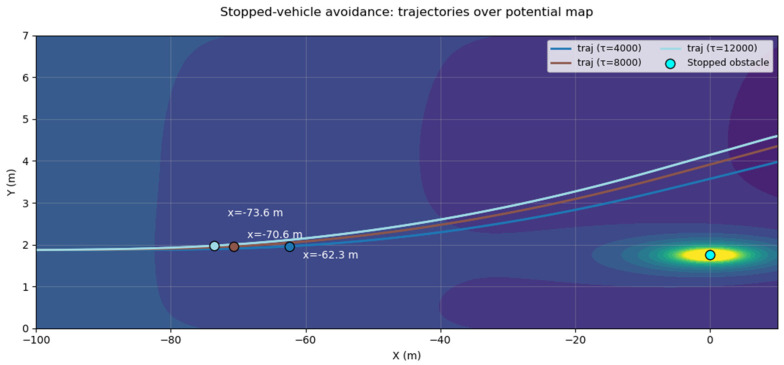
Trajectory variation and lane-change initiation distance under different τ values during stationary obstacle avoidance.

Through stationary-vehicle avoidance experiments, variations in trajectories were analyzed for different τ values, and an effective value was selected based on observed performance. Specifically, for τ = 8000, the onset of avoidance occurred at a Time To Collision(TTC) ≈ 4.2 s, which is consistent with commonly accepted safety thresholds (typically 3–5 s), as illustrated in Figure 4, which shows the trajectory variation and lane-change initiation distance under different τ values during stationary obstacle avoidance. Consequently, this value was adopted in this study.

The parameter $k_{v}$ acts as a sensitivity adjustment factor with respect to speed; higher values amplify the growth of avoidance initiation distance as speed increases, effectively reflecting realistic increases in reaction time and deceleration requirements. Conversely, $\varepsilon_{1}$ governs the decay rate of the potential with respect to distance, with larger values extending the influence of surrounding vehicles over longer ranges.

**Figure 5:**
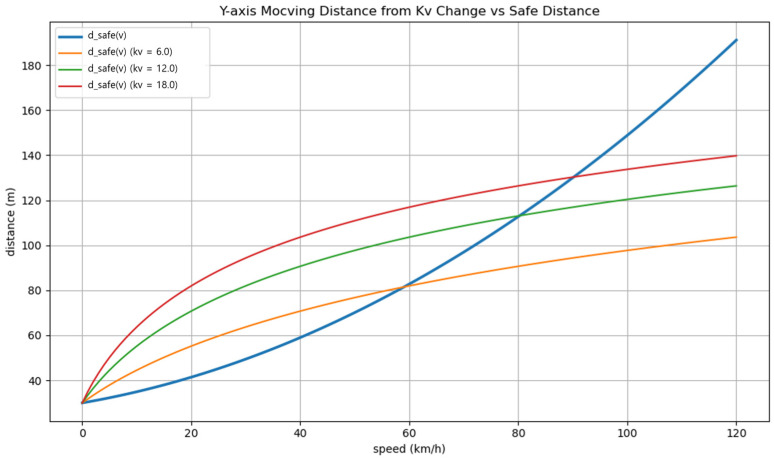
Comparison between speed-safety distance curve and potential-based risk threshold growth for different $k_{v}$ values.

As illustrated in Figure 5, $k_{v}$ = 6 provides adequate safety in the low-speed region but fails to deliver sufficient responsiveness at higher speeds due to its low growth rate relative to the safety-distance model. Conversely, $k_{v}$ = 18 ensures robust responsiveness even at high speeds, but its excessive growth at low speeds induces unnecessarily conservative behavior. In contrast, $k_{v}$ = 12 offers a balanced trade-off, closely aligning with the safety-distance curve across a typical driving speed range (0–80 km/h), intersecting near 80 km/h. This alignment secures adequate safety margins in low- and medium-speed conditions while maintaining acceptable responsiveness in high-speed scenarios. Based on these observations, $k_{v}$ = 12 was adopted as it harmonizes the potential function’s sensitivity with safety-distance standards while mitigating both overly defensive maneuvers and underreaction during real-world driving.

Other coefficients, such as the lateral-to-longitudinal influence ratio [16] and the weighting factors for lane and boundary potentials [18], were set by referencing prior literature and adjusted empirically to ensure that the vehicle remains centered within its lane under normal conditions while initiating a lane change upon exceeding predefined thresholds. Although these configurations do not represent an optimized solution, they successfully induce driving behavior within an acceptable range when compared against established safety-distance models, and they provide practically viable parameter values for experimental validation. Future work will pursue systematic parameter optimization to further enhance both safety and operational efficiency.

### 3.3. Path Generation

#### 3.3.1. Principle of GT-PF Path Generation Based on Gradient Descent

In PF-based path planning, the direction and magnitude of a vehicle’s movement are determined by gradient descent. Gradient descent is a representative optimization algorithm that searches for the minimum of a function by iteratively moving in the direction of the steepest descent—that is, the negative gradient. When applied to PF-based path planning, this method enables a vehicle to avoid regions of high potential (risk) by moving in the direction where the potential value decreases most rapidly.

This concept can be mathematically expressed as

(8) $$F\left(p\right)=-\nabla U(p)$$

Here, $F\left(p\right)$ denotes the motion direction vector, U(p) is the potential (risk) at position, and ∇U(p) represents the gradient of the potential function. In each step, the vehicle moves a fixed distance in the direction where the potential decreases most rapidly.

In practical implementations, the analytical form of the potential gradient is often unavailable, so numerical differentiation using central difference is applied. Given a small step size h, the potential change in both directions is calculated to approximate the gradient. This method can be applied not only in 2D (x-y plane) but also in time-extended spatiotemporal 3D domains (x, y, t) environments that include the temporal axis.

Although gradient descent does not guarantee a globally optimal path, it allows for real-time and responsive path generation using only local information. If the PF and its gradient are properly designed, the vehicle can efficiently avoid hazards like dynamic obstacles, complex road environments, and leading vehicles while reaching its destination.

#### 3.3.2. GT-PF: Gradient-Based Path Search in a Time-Extended Spatiotemporal Field

This study generates paths based on the GT-PF framework, which constructs a time-extended spatiotemporal potential field where both space (x, y) and time (t) are included as search variables. Within this 3D field, each vehicle position is represented as a spatiotemporal coordinate (x, y, t). This structure is advantageous over traditional 2D spatial fields because it accounts for changes over time in the environment and behavior of leading vehicles, making it suitable for dynamic situations.

To implement this in real road scenarios, it is necessary to search space and time simultaneously. However, since spatial coordinates (x, y) are in meters and time (t) is in seconds, this unit inconsistency complicates calculations of distance or displacement.

To resolve this, a spatiotemporal scaling factor α is introduced, which aligns time increments with spatial ones based on a vehicle’s desired speed. With this, space and time can be treated on the same scale during the path search, allowing the vehicle to plan a trajectory aligned with its preferred velocity.

Using this, the unit spatiotemporal distance s between two points $p_{k}=(x_{k},y_{k},t_{k})$ and $p_{k+1}=(x_{k+1},y_{k+1},t_{k+1})$, ∆s is defined as

(9) $$∆s=\sqrt{{\left(x_{k+1}-x_{k}\right)}^{2}+{\left(y_{k+1}-y_{k}\right)}^{2}{+\alpha^{2}(t_{k+1}-t_{k})}^{2}}$$

Here, α acts as the velocity scaling factor that aligns the time and space units. When the slope of the x-y plane and the t-axis is 1 (i.e., 45°), the vehicle travels at its desired velocity. Thus, when Δt = 1, the spatial movement is α, and vice versa. Given this formulation, the next spatiotemporal position is computed by:

(10) $$p_{k+1}=p_{k}+∆s\cdot d_{k}$$

where $d_{k}$ is the normalized direction vector that indicates the direction of descent in the PF from the current point $p_{k}$. It is derived as

(11) $$d_{k}=-\frac{\nabla U\left(p_{k}\right)}{\left|\nabla U\left(p_{k}\right)\right|},\;\;\nabla U(p_{k})\approx \frac{\nabla U\left(p_{k}+h\right)-\nabla U(p_{k}-h)}{2h}$$

where is determined by the resolution parameter. Smaller h gives finer gradients but may increase computational cost and sensitivity to noise.

**Figure 6:**
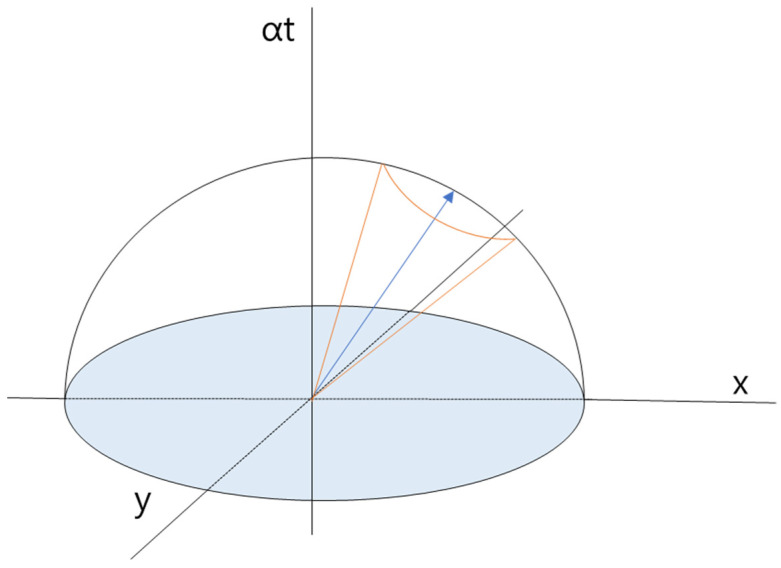
Search region of 3D spatiotemporal exploration.

When the slope in the spatiotemporal domain is 1 (i.e., 45°), the vehicle maintains its target velocity. At the start of path generation, the reachable domain forms a spherical shape centered at the initial point. By excluding backward time progression, this region becomes hemispherical. Further restricting the movement to a conical region using angular constraints enables control over speed and steering angle, as illustrated in Figure 6, which visualizes the hemispherical and conical search regions in the spatiotemporal domain.

Table 2 below shows the minimum and maximum achievable velocities when maintaining constant x-y motion under different angular limits, assuming the initial velocity v = 16.66 m/s:

Thus, angular constraints alone allow for physically natural regulation of speed and direction changes, effectively suppressing sudden acceleration or deceleration. However, if minor speed variations accumulate over time, the actual vehicle speed may deviate significantly from the desired velocity. To address this issue, a soft bias toward the desired speed α is introduced during path generation. This bias acts as a corrective mechanism to prevent excessive deviation from the target speed.

Specifically, a weight is added based on the dot product between the unit gradient vector $g_{k}$ and the unit direction vector of desired motion *v*’. When the vehicle’s current speed $v_{k}$ deviates beyond a threshold from α, the descent direction $d_{k}$ is modified as

(12) $$d_{k}=g_{k}\pm (\alpha -v_{k})\mathrm{if}\;\left|v_{k}-\alpha \right|>threshold$$

**Figure 7:**
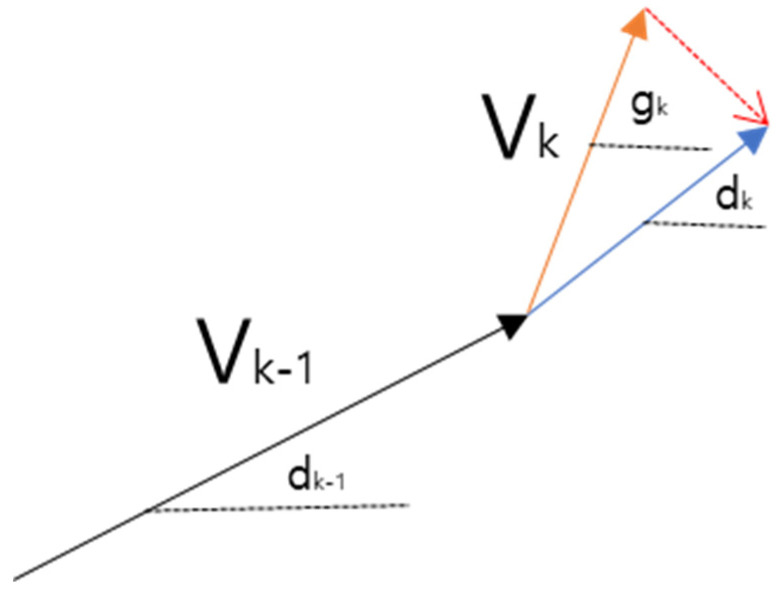
Velocity vector correction toward desired speed.

As illustrated in Figure 7, this mechanism helps steer the vehicle toward maintaining a speed trajectory that is closer to the desired velocity. The strength of this correction depends on the magnitude of the deviation $\alpha -v_{k}$ allowing adaptive control without abrupt changes.

In summary, this study proposes the GT-PF method, a time-extended spatiotemporal potential field with velocity bias technique for realistic trajectory generation. Since GT-PF provides mission-level reference paths rather than direct control commands, strict feasibility constraints are embedded at the generation stage itself, ensuring that the resulting trajectories remain physically realizable without relying on subsequent controller-based correction. In the next chapter, the method’s performance is evaluated via simulation and compared to classical sampling-based techniques (e.g., RRT) in terms of path efficiency, motion smoothness, and risk avoidance.

## 4. Simulation and Performance Evaluation

### 4.1. Implementation and Experimental Setup

In this study, the simulation environment assumes a one-way, two-lane straight road segment with a total width of 7 m. The ego vehicle starts at position x = −75 m, y = 1.75 m (center of the original driving lane) with an initial velocity of 16.66 m/s (approximately 60 km/h). The leading vehicle begins at x = 0 m, y = 1.75 m with an initial velocity of 11.11 m/s (approximately 40 km/h) and an acceleration of –0.1 m/s^2^.

**Figure 8:**
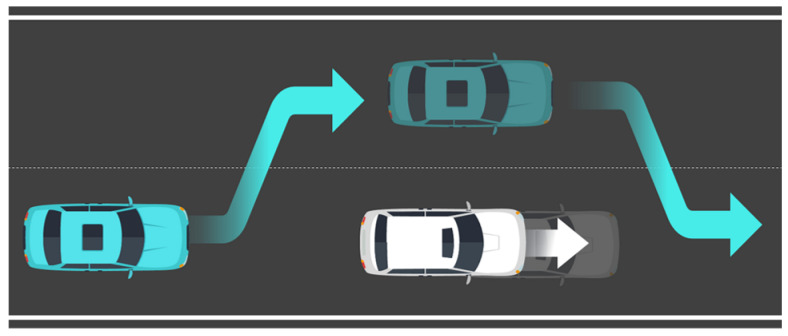
Schematic illustration of the overtaking maneuver, showing the ego vehicle’s lane-change trajectory, including the initial lane-change to the overtaking lane, acceleration to pass the leading vehicle, and subsequent return to the original driving lane.

The goal of the ego vehicle is to safely overtake the leading vehicle by changing lanes to the adjacent overtaking lane and accelerating, then returning to the original driving lane and reaching the target point at x = 500 m, y = 1.75 m. The road is divided into an original driving lane (y = 3.5 m to 7.0 m) and an overtaking lane (y = 0 m to 3.5 m). Both vehicles initially start from the center of the overtaking lane (y = 1.75 m). During the overtaking maneuver, the ego vehicle moves laterally to y > 3.5 m and then returns to approximately y ≈ 1.75 m after reentry, as illustrated in Figure 8. All driving environment information (lane boundaries, lane centerlines, and the real-time status of the leading vehicle) is assumed to be provided by a Road Side Unit (RSU) in real time, and the numerical parameters used in this simulation are summarized in Table 3.

**Figure 9:**
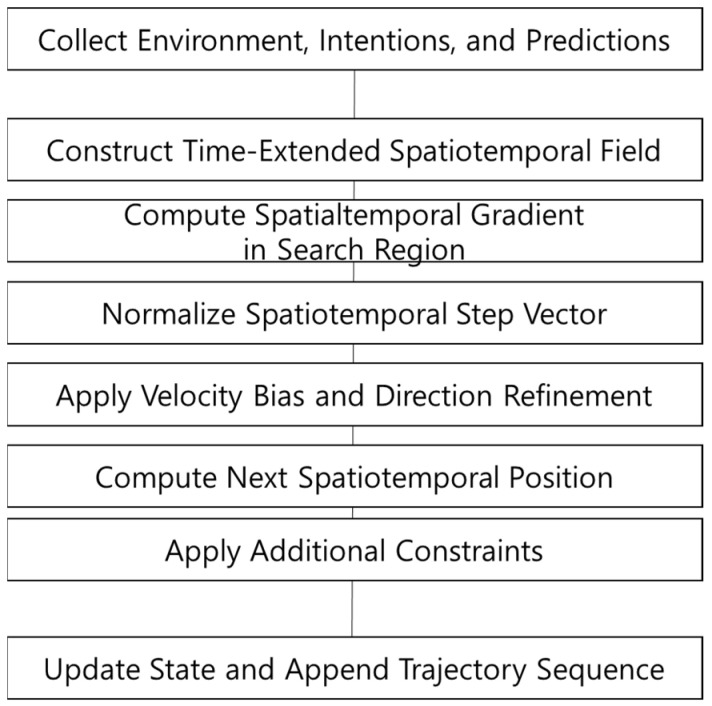
Stepwise construction of trajectory via gradient descent in the GT-PF.

This study implements and evaluates the GT-PF algorithm proposed in Chapter 3, which constructs a trajectory by following the negative gradient of a time-extended spatiotemporal potential field. The method accounts for vehicle dynamics through a time-to-distance scaling factor based on the desired speed, allowing for the generation of physically feasible and policy-compliant paths without reliance on random sampling. Figure 9 provides a schematic flow of this GT-PF path planning process, illustrating the step-by-step trajectory construction over a time-extended potential landscape.

For performance comparison, a Rapidly exploring Random Tree (RRT) approach was implemented under the same time-extended spatiotemporal potential field structure. The choice of RRT is motivated by prior studies that applied RRT-based path planning within similar spatiotemporal potential field, making it a suitable benchmark to assess the practical advantages of the proposed method [36]. The RRT implementation in this study employed random sampling with an additional goal-bias strategy, where the goal point was sampled with a 10% probability to improve convergence. Sampling was performed in the three-dimensional (x, y, t) space, and a fixed random seed was used to ensure reproducibility. Advanced sampling techniques (e.g., informed or heuristic bias) were not applied; instead, correction based on the gradient of the Spatiotemporal Risk Field (STRF) was integrated into the extension step to enhance safety margins and exploration stability.

Tree extension proceeded from the nearest node toward the sampled point, with spatial steps limited to a maximum of 8 m and temporal steps limited to a maximum of 0.5 s. The temporal node resolution itself was aligned with GT-PF at 0.01 s, ensuring consistent comparison. The nearest node was determined using a composite metric that considered position difference, the speed and acceleration of the leading vehicle, and STRF energy difference, enabling simultaneous consideration of time, dynamics, and safety. For each new node, a STRF gradient-based correction was applied to guide the exploration toward safer zones within the PF rather than purely random expansion.

The maximum number of nodes was set to 1000, chosen as a practical trade-off between spatial resolution and computational cost. Preliminary tests revealed that fewer than 500 nodes resulted in unstable collision avoidance and poor path continuity, whereas more than 2000 nodes significantly increased computation time with only marginal improvements in path quality. Thus, the 1000-node limit ensured sufficient exploration fidelity and served as a representative baseline against which the GT-PF approach could be assessed under real-time constraints.

While RRT provides flexibility and probabilistic completeness, it typically incurs higher computation time and sensitivity to sampling configuration. The subsequent experiments aim to examine how the proposed GT-PF method performs relative to this representative sampling-based approach in terms of safety, efficiency, and real-time feasibility in cooperative driving environments.

The evaluation metrics used for quantitative assessment of the path generation algorithms are summarized in Table 4. All metrics are derived under identical conditions to ensure consistent comparison. Section 4.2.2 presents result by algorithm and time resolution, enabling comprehensive analysis of path generation quality, safety, and computational efficiency. The simulation framework, including the proposed GT-PF method algorithm and the baseline RRT method, was implemented in Python 3.11.5 All experiments were conducted on a standard desktop environment (Intel Core i7-12700 CPU, 16 GB RAM), without GPU acceleration. While sufficient for simulation purposes, the current implementation has not been optimized for computational efficiency. In real-world deployment, the available processing power of RSU is typically more limited. Therefore, further code optimization and lightweight implementation strategies will be essential for practical integration into infrastructure-based cooperative driving systems.

### 4.2. Comparative Results and Discussion

#### 4.2.1. Path Visualization and Dynamic Characteristics

**Figure 10:**
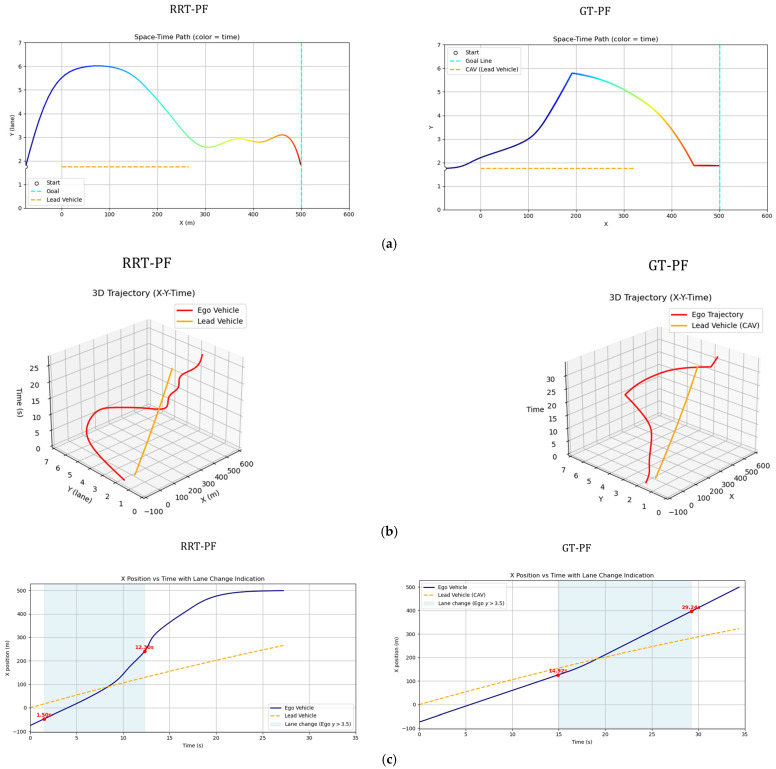
Spatiotemporal trajectories of ego and lead vehicles: (**a**) top-down view of the vehicle trajectories in the spatial domain (x-y plane); (**b**) spatiotemporal paths represented in a three-dimensional space (x-y-t); (**c**) time–distance plot(t-x).

Figure 8 illustrates the overtaking trajectories generated by both methods, along with their corresponding temporal and behavioral characteristics. In Figure 10a, the gradient-colored curved line represents the trajectory of the ego vehicle, while the orange line shows the path of the leading vehicle.

In the sampling-based RRT method, the ego vehicle initiates a lane change almost immediately and attempts to return to the original lane shortly after overtaking. However, it does not maintain a central position within the lane and appears to be pulled toward the final goal near the destination due to the attractive potential. Because the RRT method is sensitive to the distribution of sample nodes, slight oscillations may occur near the goal as the vehicle proceeds in a nearly straight line. In contrast, the GT-PF method keeps the ego vehicle aligned with the lane center for a longer duration before initiating the lane change. After completing the overtaking maneuver, it promptly returns to the original lane and continues its trajectory steadily toward the destination. Figure 10b provides the time-aligned visualization of these paths, indicating that the leading vehicle moves with constant speed and acceleration, while the ego vehicle shows minor velocity variation but maintains an overall consistent progression. Figure 10c highlights the timing of key maneuvers. The lane change in the GT-PF method occurs more gradually, around 14 s, while the RRT-based method completes the lane change earlier, around 2 s. The overtaking itself takes place at approximately 16 s for the GT-PF method and around 8 s for the RRT method. Both vehicles subsequently return to the original lane and continue toward the goal.

**Figure 11:**
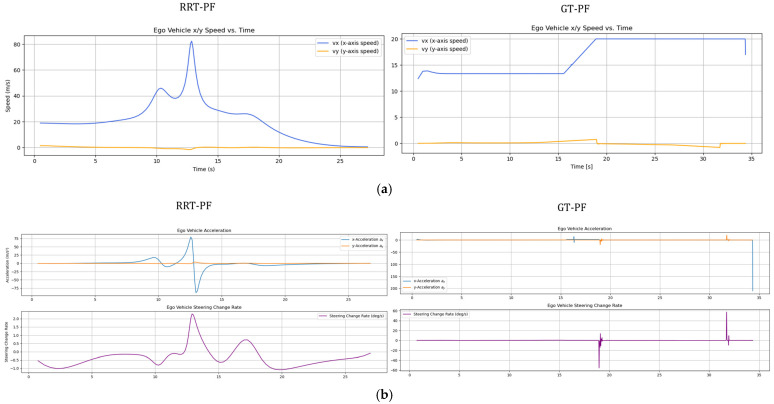
Velocity, acceleration, and steering variation by experiment: (**a**) longitudinal and lateral velocity profiles; (**b**) acceleration and steering angle variation.

Figure 11a presents the longitudinal and lateral velocity profiles. The RRT method shows abrupt speed changes in certain segments, indicating discontinuities in the path due to irregular sample connections. On the other hand, the GT-PF method generally adheres to the recommended velocity at the beginning but later maintains a slightly higher speed after overtaking and lane reentry, possibly to ensure sufficient separation from the leading vehicle. Figure 11b illustrates acceleration and steering dynamics during the path traversal. In the RRT-based method, frequent accelerations and steering adjustments occur in response to abrupt changes in path direction. The GT-PF method exhibits more stable acceleration and steering behavior overall, although sharp changes occasionally arise—particularly when the ego vehicle attempts to stay aligned with the center of the lane.

Both approaches enable the ego vehicle to successfully reach the destination ahead of the leading vehicle by performing an overtaking maneuver. However, they exhibit distinct differences in terms of lane change timing, acceleration and deceleration profiles, and steering behavior. These observations highlight the characteristic planning tendencies of each method. To further assess their practical implications, the following section presents a quantitative evaluation focusing on safety, efficiency, comfort, and computational performance.

#### 4.2.2. Quantitative Comparison

The results of the quantitative evaluation demonstrate that the GT-PF method has clear advantages over RRT in terms of computational efficiency, dynamic comfort, and average potential risk across the entire trajectory, as shown in Table 5.

First, the computation time of the GT-PF method is only 0.35 s, compared to 10.89 s for RRT—a reduction of approximately 97%, indicating strong suitability for real-time applications. The total travel distance is nearly identical for both methods. However, by reducing average acceleration by 94% and lowering maximum jerk by over 64%, the GT-PF method generates significantly smoother and more dynamically safe paths, enhancing ride comfort throughout the maneuver. In particular, during the final cruising phase after completing the lane return maneuver, the GT-PF method consistently generates stable and center-aligned trajectories. This is because, in the absence of interfering potential sources, the lane potential produces a local minimum along the centerline, and the resulting gradient naturally guides the vehicle along that path. In contrast, while RRT tends to sample nodes closer to the lane center due to potential-based cost minimization, the actual distribution depends heavily on random sampling density. Although post-processing techniques smooth the resulting path, they often fall short of maintaining a consistent centerline trajectory throughout the lane.

Moreover, the average risk per meter is 40% lower with the GT-PF method, indicating better avoidance of sections with high cumulative potential values throughout the trajectory. This reflects the method’s consistent tendency to steer away from risk-prone regions by following the negative gradient of the PF and adhering to strict dynamic constraints. Even when passing through locally risky areas becomes momentarily unavoidable, the overall trajectory remains globally safer. On the other hand, RRT—despite being capable of finding local minima in cost function and avoiding extreme peaks around specific nodes—has a structural limitation in assessing the cumulative safety of the entire path formed by connecting multiple nodes. In our implementation, we attempted to address this by integrating potential-based gradient corrections, but the tree-based expansion mechanism inherently limits its ability to account for region-wide safety optimization.

The GT-PF method does have some performance trade-offs. The arrival time is approximately 26% longer, risk-zone dwell time increases by around 103%, and the minimum safety distance is 17% shorter compared to RRT. These drawbacks are attributable to the limited exploration and absence of backtracking in the GT-PF framework. While this results in significantly reduced computational cost and improved stability, it limits the method’s ability to discover globally optimal or more aggressive paths. Nonetheless, this tendency aligns with the design goal of GT-PF, which is to generate dynamically feasible and stable local trajectories rather than perform exhaustive global searches. Therefore, the method is well-suited for applications that emphasize local path planning or adjustment rather than global optimality across the entire trajectory.

Although localized behaviors—such as reduced minimum clearance or extended risk-zone dwell times compared to RRT-PF—were observed, additional verification confirmed that the trajectories remained within acceptable operational limits. The GT-PF framework is fundamentally designed to enforce safety through repulsive potentials that inherently discourage unsafe proximity and maintain lane compliance, meaning that these variations do not indicate any collision risk or hazardous maneuver. Nevertheless, to provide objective assurance, post hoc validation was performed using widely recognized safety indicators under dynamic traffic conditions.

**Figure 12:**
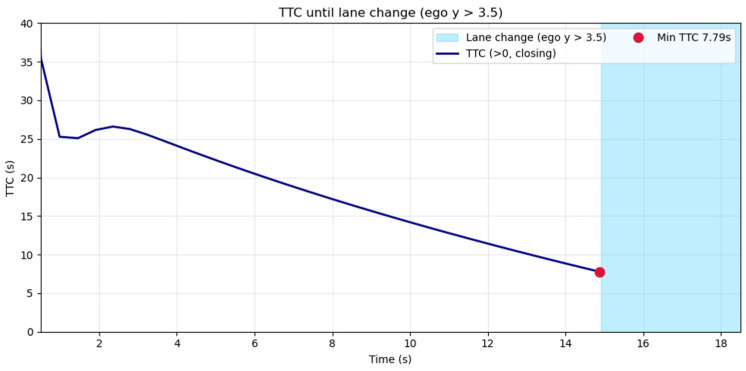
TTC during the car-following phase prior to lane-change initiation.

Specifically, TTC, defined as τ = −r/r′, was applied during the initial car-following phase and consistently exceeded 7 s, reaching a minimum of 7.8 s immediately before the lane-change maneuver (t ≈ 14.9 s), well above the commonly cited threshold of 4–5 s (Figure 12). This gradual decrease without abrupt drops indicates sufficient buffer for maneuvering without emergency braking.

**Figure 13:**
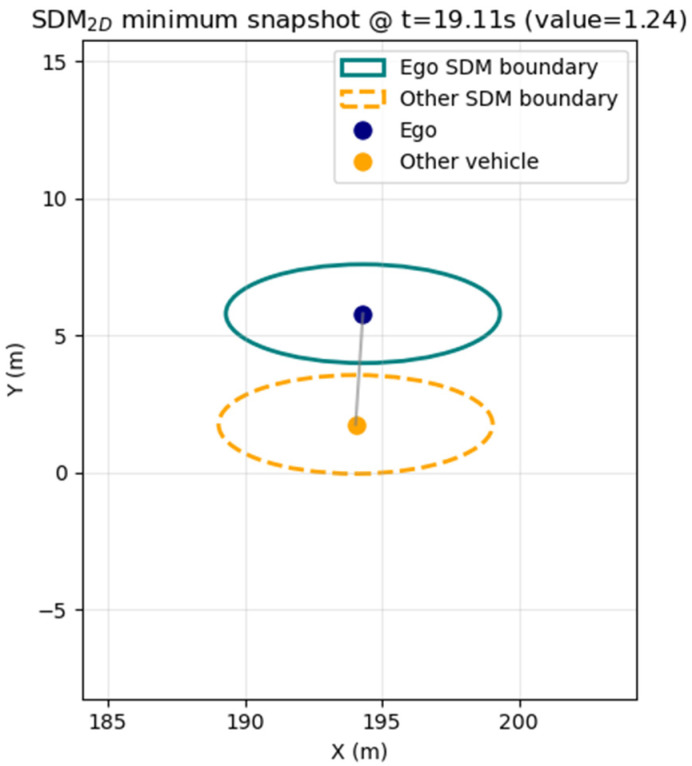
Snapshot of safety buffer at the closest interaction point (ellipses for visualization; not actual vehicle size).

After the lane-change initiation, TTC becomes less applicable because the vehicles occupy different lanes; therefore, we employed the Safety Distance Margin (SDM) as a complementary metric. SDM was computed using a Minkowski-sum-based approach widely used in robotic motion planning [40], where collision checking is simplified by representing two vehicles as a single expanded ellipse rather than computing the intersection of two separate ellipses. In the schematic (Figure 13), both vehicles are depicted as individual ellipses for visualization purposes; however, the actual computation uses one enlarged ellipse that combines both vehicles’ dimensions and safety margins. The safety buffer was defined by vehicle dimensions plus minimum safety margins (lateral 1.5–2.0 m, longitudinal 4.0–5.0 m). Throughout the simulation, SDM remained positive, with the minimum value (≈ 1.24) observed near t ≈ 19 s when longitudinal positions overlapped across adjacent lanes, confirming that both vehicles maintained non-intersecting safety envelopes even during the closest interaction.

Although GT-PF-based indices indicated transient increases in local risk, the combined evidence from TTC, SDM, and the decreasing trend of average potential along the trajectory demonstrates that the maneuver maintained an operationally acceptable safety level. These findings suggest that GT-PF can ensure sufficient clearance while delivering smoother and computationally efficient trajectories, even when local safety margins fluctuate.

In conclusion, the GT-PF-based approach is better suited for cooperative infrastructure-based driving systems, where real-time performance, comfort, and risk minimization are prioritized. While certain local safety indicators showed temporary reductions, these remained within acceptable thresholds, confirming that the method ensures operational safety even under such fluctuations. However, for applications that require rapid arrival, shortest path, or aggressive avoidance in complex environments, the RRT-based method may still offer advantages. It should be noted that the simulation was conducted on a desktop environment, and the computation time of 0.35 s for the GT-PF-based method reflects such conditions. In real-world deployments, infrastructure-based systems such as RSUs or MEC (Multi-access Edge Computing) units may operate with more limited computational resources. Nonetheless, the simplicity and computational efficiency of the gradient approach suggest its promising adaptability to such environments. With lightweight optimization and parallel processing, this method can serve as a viable real-time path planning solution within cooperative infrastructure systems, even under constrained edge computing conditions.

#### 4.2.3. Parameter Sensitivity

To examine the generalizability of the proposed path planning method, we conducted supplementary simulations under several modified initial conditions beyond the primary test scenario. These tests aimed to qualitatively verify the adaptability and safety of the GT-PF method in diverse driving environments.

**Figure 14:**
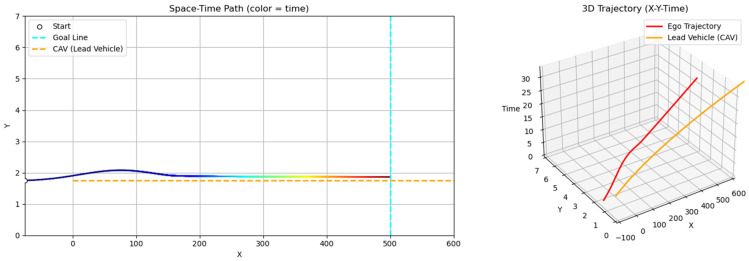
Scenario A: Following without overtaking (both 60 km/h).

Scenario A considers both the ego vehicle and the lead vehicle departing at the same speed (60 km/h), with the lead vehicle maintaining a constant velocity throughout. In this case, the ego vehicle follows the lead vehicle without overtaking, maintaining a safe distance near the lane center and reaching the destination after the lead vehicle exits the scene (Figure 14). This scenario illustrates the GT-PF method’s ability to determine whether overtaking is necessary. When smooth following is feasible without excessive deceleration, the ego vehicle maintains a proper following distance and continues lane-keeping rather than initiating unnecessary lane changes, guided by dynamic risk assessment. In addition, the ego vehicle consistently tracks the lane centerline during the following phase, demonstrating the method’s effectiveness in stable lane-keeping under low-risk conditions.

**Figure 15:**
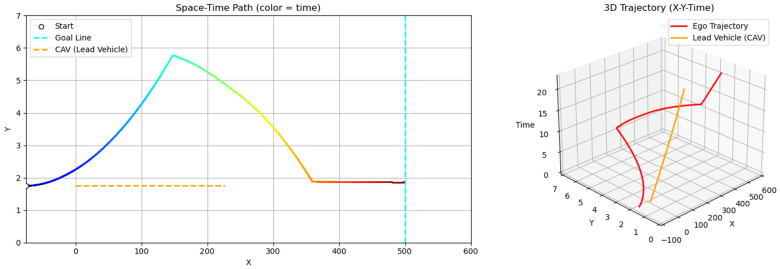
Scenario B: early overtaking (ego: 80 km/h, lead: 40 km/h).

**Figure 16:**
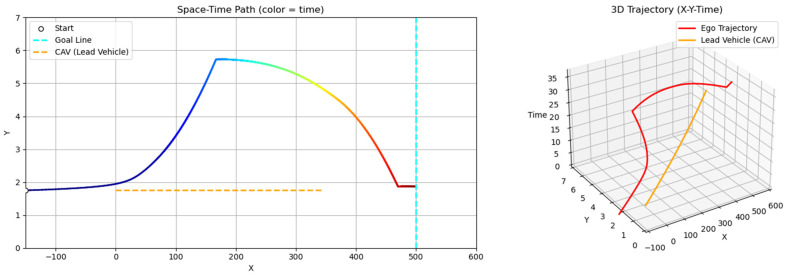
Scenario C: delayed overtaking (longer initial gap: 150 m).

Scenario B examines a situation where the ego vehicle departs at a significantly higher speed (80 km/h) than the slower lead vehicle (40 km/h). As a result, the ego initiates an immediate lane change maneuver to overtake, without a preceding following phase (Figure 15). Scenario C increases the initial spacing between vehicles: the ego vehicle departs from 150 m behind the lead vehicle (compared to 75 m in the main case). In this case, the ego vehicle travels along the lane centerline for a longer duration and initiates a lane change only when entering the zone of elevated risk influenced by the lead vehicle (Figure 16). These two scenarios provide insight into the GT-PF method’s capability to determine the appropriate timing for overtaking. The ego vehicle does not initiate overtaking simply due to the presence of a slower lead vehicle; instead, it performs such maneuvers only when the level of risk increases to the point where maintaining a safe following distance is no longer viable.

Together, these variations demonstrate that the GT-PF method adapts to different speed and spacing conditions by generating context-appropriate trajectories—whether maintaining lane centerline tracking or initiating overtaking as required. The method effectively balances safe following and proactive maneuvering by evaluating risk dynamically. These observations confirm that the proposed time-extended potential field approach remains broadly applicable across diverse driving situations, including lane-keeping, overtaking, and following. While it may not capture every edge case, it shows robust responsiveness and adaptability to a wide range of realistic traffic conditions.

#### 4.2.4. Extended Scenario

The primary scenario employed in this study involves a single leading vehicle; however, it inherently encompasses diverse driving behaviors, including initial car-following, lane changing, parallel lane driving, overtaking, and front merging. These characteristics enable the scenario to serve as a comprehensive testbed, allowing the proposed method to be validated under multiple behavioral contexts despite being a single case.

Nevertheless, real-world traffic conditions frequently involve non-uniform maneuvers of the leading vehicle or interactions with multiple surrounding vehicles. To ensure that the proposed method does not overfit a specific scenario and maintains robustness across varied traffic situations, additional sub-scenarios were designed. These scenarios introduce variations in leading vehicle behavior and the presence of multiple vehicles, thereby allowing the assessment of trajectory continuity, collision avoidance capability, and stability of spatiotemporal planning under diverse conditions.

**Figure 17:**
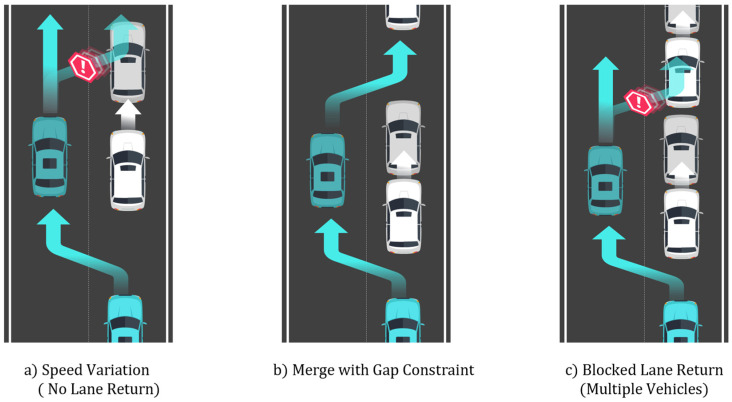
Additional sub-scenarios for evaluating the proposed method: (**a**) speed variation with no lane return; (**b**) merge with gap constraint; and (**c**) blocked lane return involving multiple vehicles.

Figure 17 illustrates the sub-scenarios designed in this study. These sub-scenarios were developed to reflect conditions where the leading vehicle exhibits non-uniform behavior or where multiple vehicles interact within the traffic environment. Sub-Scenario A represents a case where due to fluctuations in the leading vehicle’s speed, returning to the original lane after overtaking becomes hazardous, resulting in continued driving in the adjacent lane. Sub-Scenario B models a situation where the ego vehicle returns to the original lane by merging between two vehicles, requiring careful gap acceptance. Sub-Scenario C considers a more complex multi-vehicle in which another vehicle occupies the original lane during the intended return, causing a delay or preventing the maneuver entirely. These sub-scenarios are introduced to assess whether the proposed method can generate safe and adaptive trajectories under diverse and dynamic traffic conditions.

**Figure 18:**
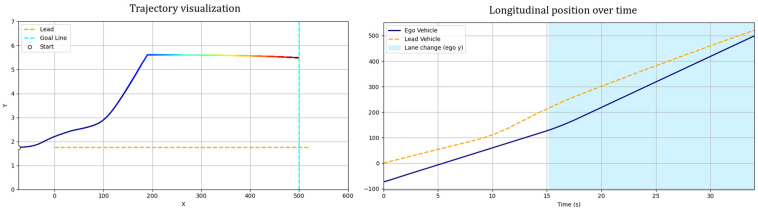
Sub-Scenario A: speed variation (no lane return).

Sub-Scenario A assumes a situation where after a lane change due to a slow vehicle in the initial lane, the leading vehicle accelerates after a certain period and maintains a speed similar to that of the ego vehicle (Figure 18). Under these conditions, returning to the original lane may be unnecessary or even hazardous. Consequently, the ego vehicle adopts a strategy of maintaining stable driving in the center of the current lane without returning to its previous lane.

This scenario does not involve a fixed trajectory of changing lanes and then returning; rather, it evaluates the capability to readjust the trajectory in response to situational changes. In real-world traffic environments, leading vehicles do not maintain a constant speed or uniform acceleration but often exhibit various speed variations during driving. Therefore, including this scenario demonstrates that the proposed method operates reliably under conditions involving speed fluctuations, not merely constant-speed assumptions.

Simulation results indicated that the ego vehicle responded appropriately to the acceleration of the leading vehicle, refraining from returning to the original lane and maintaining stable lane-centered driving instead. This suggests that the proposed path planning algorithm possesses the flexibility to adapt effectively to non-uniform speed conditions. Unlike conventional approaches that adhere to a predefined lane-return objective, the proposed method selects a risk-minimization strategy based on situational dynamics to prevent collisions.

**Figure 19:**
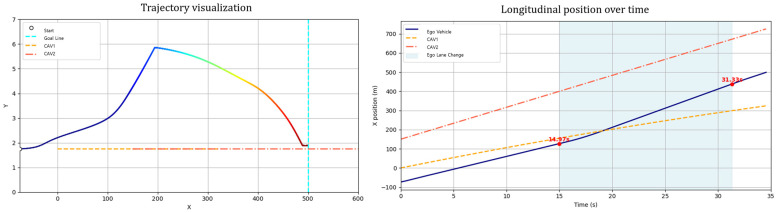
Sub-Scenario B: merge with gap constraint.

Sub-Scenario B assumes the presence of two vehicles ahead, where the leading vehicle travels at a sufficiently high speed, and the inter-vehicle gap is adequately large (Figure 19). Under such conditions, the ego vehicle recognizes an opportunity to safely merge between the two vehicles, and the proposed path planning method decides to execute the merging maneuver.

Simulation results demonstrated that the ego vehicle successfully merged between the two vehicles and subsequently maintained an appropriate longitudinal gap while continuing to drive stably. This indicates that the proposed approach is effective not only for simple overtaking or single-lane-change maneuvers targeting an individual leading vehicle but also for complex environments where multiple vehicles coexist. In particular, the PF-based structure reflects the interactions among multiple vehicles, thereby achieving simultaneous objectives of maintaining safe inter-vehicle distances and avoiding collisions.

**Figure 20:**
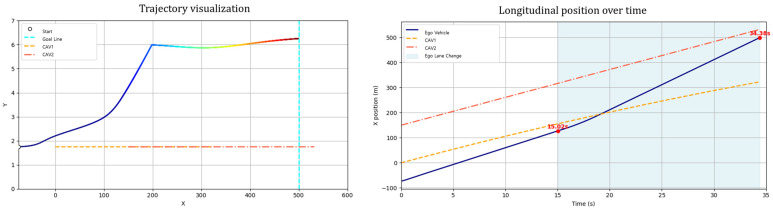
Sub-Scenario C: blocked lane return (multiple vehicles).

Sub-Scenario C considers a condition in which two vehicles are present ahead, but due to the low speed of the foremost vehicle and the limited spacing between them, merging between the two is deemed unnecessary or unsafe for maintaining driving stability (Figure 20). Consequently, the ego vehicle refrains from attempting to merge and instead adopts a waiting strategy in its original lane.

Simulation results showed that the ego vehicle avoided unnecessary lane changes, maintained a stable inter-vehicle distance, and continued traveling in the original lane. This outcome demonstrates that the proposed path planning algorithm does not mechanically attempt merging but rather makes context-aware decisions that prioritize safety. This scenario serves as an evaluation of the method’s ability to flexibly reconfigure plans and avoid risky maneuvers in complex traffic conditions.

The proposed approach was initially designed and implemented based on a single primary scenario; however, the additional experiments described above validate its stability and adaptability under diverse traffic conditions. The computation times for Sub-Scenarios A, B, and C were approximately 0.37 s, 0.38 s, and 0.41 s, respectively, demonstrating minimal variation across different traffic complexities. These findings confirm that the algorithm can effectively adjust the trajectory in response to dynamic factors, such as variations in the speed of leading vehicles, multi-vehicle interactions, and the feasibility of merging opportunities. Specifically, the method exhibits the capacity to flexibly recalibrate path shapes and optimize lane-change timing and lane-return decisions according to situational demands. Nevertheless, this study is limited to straight road segments, and further work is required to address complex environments involving intersections, curves, and variable lane configurations.

#### 4.2.5. Impact of Communication Latency on Path Planning

This study addresses a path-planning architecture that operates at the infrastructure edge unit by leveraging vehicle information transmitted via cooperative messages. Within this structure, system performance is highly dependent on communication quality. Latency or packet loss can cause discrepancies between perceived and actual motion states of leading vehicles. Despite this, the system maintains robustness because the GT-PF framework inherently drives motion toward safe zones while preserving sufficient clearance, and the GT-PF vehicle potential function incorporates speed- and acceleration-dependent repulsion. As a result, even when outdated state information is used, the resulting trajectories remain within an acceptable safety margin. A simulation scenario was configured to validate system robustness under degraded communication conditions. No predictive compensation was applied; instead, the system continued planning based on the most recently received state, regardless of its delay. The test environment imposed an average latency of 0.5 s with jitter of ± 0.1 s and a packet loss probability of 20%, which represents a conservative and highly challenging condition compared to typical cooperative message update intervals of approximately 0.1 s. These settings allow verification of whether the proposed approach can maintain operational safety under severe and realistically adverse communication conditions.

**Figure 21:**
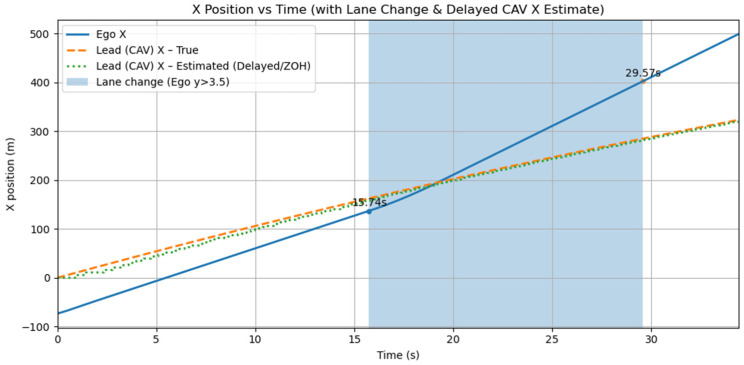
x-t plot of actual and delayed leading vehicle trajectories with ego response under latency.

Figure 21 illustrates the discrepancy between the actual vehicle position and its delayed perception due to communication latency, along with the ego vehicle trajectory under these conditions. Table 6 summarizes the comparative results against the ideal scenario without latency.

The simulation results indicate a marginal increase in arrival time by 0.17%, while the minimum gap to the leading vehicle decreased by up to 7.92%. Despite these deviations, the overall trajectory behavior remained comparable to the zero-latency case, demonstrating the inherent robustness of the GT-PF approach. However, it should be noted that these results were obtained under a single operational context with delays randomly distributed based on a normal distribution around the specified average latency, and may not fully represent extreme or irregular communication conditions. Nevertheless, given that typical cooperative message update intervals are around 0.1 s, the applied average latency of 0.5 s represents a highly conservative scenario, reinforcing the robustness of the evaluation.

Future research could explore predictive compensation mechanisms, such as equivalent acceleration-based state estimation, to interpolate missing or delayed data, thereby enhancing system resilience. Although such strategies hold significant potential, the primary focus of this study remains on validating the core infrastructure-assisted path-planning framework. Consequently, the detailed design and quantitative evaluation of predictive compensation methods are reserved as future work.

Noise and unexpected maneuvers can also pose critical challenges in path planning. Noise becomes a significant consideration particularly when cooperative messages fail to be transmitted or received, or when information about CHVs (non-cooperative vehicles) must be supplemented using infrastructure-based sensing devices. Unexpected maneuvers similarly emerge as major risk factors when vehicles exhibit behaviors that deviate from their cooperative messages or when their intentions cannot be communicated, necessitating state estimation based on infrastructure observations. However, the present study focuses on vehicles that perform normal cooperation through message-based communication; thus, noise and unexpected maneuvers are expected to play a relatively minor role. Accordingly, this study concentrated on communication latency, while the issues of noise and unexpected maneuvers remain important future research directions to be addressed under environments involving empirical measurements.

## 5. Conclusions

### 5.1. Discussion of Results

This study conducted a comparative analysis of path generation characteristics and performance between the sampling-based RRT method and the GT-PF (Gradient-based Time-extended Potential Field) method within a time-extended spatiotemporal potential field framework. The experimental results showed that the RRT method, which randomly samples the entire path space, offers flexibility and potential for optimal path discovery—especially under sufficient sampling density—in scenarios involving obstacle avoidance, route branching, and complex environments. In contrast, the GT-PF method incorporates a bias toward the desired velocity and sequentially updates the path at each step along the negative gradient direction of the potential. The search range is determined by the characteristics of a time conversion coefficient derived from the desired speed, enabling realistic driving behavior such as path smoothness, speed maintenance, and compliance with acceleration and steering constraints. This structure naturally reflects driving policies.

In terms of computational efficiency, the GT-PF method demonstrated significantly faster processing times than RRT. The experiments revealed over a 90% reduction in total computation time, confirming its suitability for real-time path generation. However, due to its strict rule-following nature, the GT-PF method may have lower success rates in reaching goals in more complex environments, and it does not guarantee globally optimal paths. On the other hand, the RRT method’s path quality depends heavily on the number and distribution of sampled points. If the sample size is insufficient, the path quality deteriorates. Furthermore, RRT paths tend to be less smooth or optimal. Increasing the number of samples improves search quality but also increases computation time, making it essential to balance sample size and search efficiency.

In summary, the GT-PF method is advantageous for real-time responsiveness and adherence to driving policies, making it particularly suitable for short-term cooperative driving in real-world infrastructure environments. Conversely, while the RRT method has higher computation cost, it remains strong in global optimization, handling exceptional situations, and exploring complex scenarios. Additional simulations under modified vehicle configurations further confirmed that the GT-PF method consistently maintained trajectory characteristics aligned with driving policies, regardless of initial speed or position. To further validate robustness, additional scenarios were examined, including dynamic traffic conditions and communication imperfections. In sub-scenarios involving speed fluctuations, multi-vehicle interactions, and merging feasibility, the proposed GT-PF framework demonstrated adaptive behavior by recalibrating lane-change and return decisions in real time. Moreover, latency simulations under severe delay and packet loss conditions showed minimal degradation in safety margins and only marginal increases in arrival time, highlighting the method’s resilience within cooperative infrastructure-based environments.

### 5.2. Evaluation from the Perspective of Infrastructure Application

From the perspective of infrastructure-assisted driving with RSU-based cooperation, the GT-PF method proves to be a practical alternative for rapidly computing paths under limited computational resources. It can easily reflect each vehicle’s desired speed, policy requirements, and real-time environmental changes, while maintaining high computational efficiency. Simulations confirmed that safety indicators and travel distances were comparable to those of RRT, while computation time was significantly reduced. Notably, the GT-PF method enables effective reflection of driving strategies such as speed preference and policy adherence through simple adjustments of the time conversion coefficient and bias parameters. This makes it well suited for policy-driven path control in infrastructure-to-vehicle systems.

However, in specific situations such as proximity to obstacles, unexpected hazards, or the need for global optimization and rapid arrival, the RRT method exhibited superior performance. Therefore, in RSU-based infrastructure systems where real-time performance and computational efficiency are prioritized, the GT-PF method is realistically favorable. For scenarios requiring rapid arrival or handling of exceptional complexity, a hybrid approach using RRT selectively may be beneficial.

These findings provide a practical foundation for selecting path generation methods based on the specific environment and priorities when constructing future infrastructure-based cooperative driving systems. This approach enables RSUs to generate safe and efficient paths in real time, making it suitable for scalable deployment in public cooperative driving systems.

In addition, the robustness of the proposed GT-PF approach was evaluated under the GT-PF framework can maintain stable performance by utilizing previously received states, even when partial data loss or delay occurs. While this study focused on latency, other factors such as sensing noise and unexpected vehicle behavior remain significant challenges for infrastructure-based systems. These issues should be addressed in future work to ensure comprehensive robustness under heterogeneous traffic conditions. communication imperfections, reflecting realistic RSU-based environments. A latency scenario with severe delay (0.5 s on average), jitter (± 0.1 s), and packet loss rates of up to 20% was applied. Despite these conditions, safety margins decreased by no more than 7.9%, and arrival time increased by less than 0.2% compared to ideal communication. This demonstrates that the GT-PF method maintains robust trajectory planning under realistic communication imperfections, confirming its suitability for infrastructure-assisted cooperative driving.

### 5.3. Limitations and Future Work

This study focused on exploring the feasibility of applying PF-based path generation in infrastructure-based cooperative driving environments. Although simulation scenarios were constructed to highlight the dynamic characteristics of the proposed method, they remain relatively simple and limited compared to real-world road conditions. The experiments were conducted on a straight two-lane road with one ego vehicle and one lead vehicle, which differs from real traffic environments that involve diverse road geometries and interactions with multiple vehicles. Moreover, the performance of each method may vary depending on parameter settings and weighting factors used in the simulation. The study does not address real-world uncertainties such as sensor detection range, communication delays, or prediction errors in road conditions. Future research should include evaluations under dynamic environments with multiple vehicles and more diverse road structures (e.g., curves, intersections, ramps). To apply the methods in real cooperative systems, the gradient-based GT-PF method approach needs to be enhanced. This includes

- Further refining the integration of driving policies (e.g., steering angle change rate, vehicle following, lane selection);
- Increasing the influence of potential-based safety distances for obstacle avoidance;
- Detecting and escaping from local minima.

These improvements aim to make the path generation process more flexible and responsive to changes in the environment.

Additionally, practical implementation challenges must be addressed, such as

- Real-time applicability under RSU-to-vehicle communication systems;
- Integration of vehicle sensor data and infrastructure-provided data;
- Strategies for emergency maneuvers and handling unexpected obstacles;
- Mitigation of communication imperfections (e.g., latency, jitter, packet loss) and robustness against sensing noise.

These findings suggest that the GT-PF path planning method offers a promising balance between real-time performance and path feasibility, making it a practical foundation for future cooperative driving systems under intelligent infrastructure.

## Figures and Tables

**Table 1:** Comparison of prior PF-based methods and GT-PF.

Category	GT-PF	[30]	[36]	[39]
Agent Potential Function	velocity and acceleration aware potential	APF	APF (vehicle-type variant)	Risk Field
Exploration Method	Gradient-based PF Search	MPC-based Optimization	MPC-based Control	RRT-based Sampling
Planning Dimension	Space–Time (Gradient-based)	Space (2D)	Space + Communication	Space–Time (RRT-based)
Dynamic Adaptation Method	Time-Extended PF with Gradient Search	Real-time MPC re-optimization	Cooperative MPC with V2V/V2X	Time-Expanded RRT Sampling
Real-time Capability	High	Medium	Medium	Low (due to sampling)
Control Level	Trajectory-level (no direct control)	MPC-level (explicit control input)	MPC-level	Trajectory-level (slow, heavy)
Road & Lane Structure	Trajectory-level (no direct control)	MPC-level (explicit control input)	MPC-level	Trajectory-level (slow, heavy)
Cooperation	Infrastructure-assisted	Single	Cooperative	Connected

**Table 2:** Speed constraints based on search angle.

Search Angle	±1°	±2°	±3°	±5°	±10°	±15°	±30°
Maximum speed (m/s)	1.04v (17.25)	1.07v (17.88)	1.11v (18.50)	1.19v (19.87)	1.43v (23.81)	1.73v (28.85)	3.73v (62.18)
Minimum speed (m/s)	0.97v (16.09)	0.93v (15.54)	0.90 v (15.00)	0.84v (13.97)	0.70v (11.67)	0.58v (9.62)	0.27v (4.47)

**Table 3:** Simulation scenario parameters.

Category	Item	Value/Description
Road	Road type	Two-lane one way, Straight section
	Road width	7 m
Ego Vehicle	Initial Position	(x, y) = (−75, 1, 75)
	Initial Speed	16.66 m/s (60 km/s)
Leading Vehicle	Initial Position	(x, y) = (0, 1.75)
	Initial Speed	11.11 m/s (40 km/s)
	Initial acceleration	0.1 m/s^2^
Goal point		(x, y) = (500, 1.75)
Time resolution	∆t	0.1 s
PF/dynamics Parameter	Defined internally in the implementation; not detailed here	

**Table 4:** Evaluation Metrics for Path Planning Performance.

Metric	Definition
Arrival time and travel distance (Efficiency)	Total time and distance taken by the ego vehicle to reach its goal.
Minimum and average distance to the leading vehicle (Safety)	Minimum and average inter-vehicle distance throughout the scenario.
Time spent in risk zones (Critical Time in Risk Zone)	Cumulative time with distance to the lead vehicle less than 50 m.
Integrated potential risk (Integrated Risk)	Time-integrated potential field values, normalized by travel distance.
Acceleration and jerk (Comfort)	Maximum and average longitudinal/lateral acceleration, jerk, steering rate.
Computation time (Computational Cost)	Total processing time required to generate a full trajectory.

**Table 5:** Quantitative comparison of performance metrics between GT-PF and RRT-PF-based trajectory planning methods.

Category	RRT-PF	GT-PF	Relative Difference (%)
Arrival Time (s)	27.23	34.36	+26.18
Travel Distance (m)	574.23	572.69	−0.27
Minimum Safety Distance (m)	4.89	4.04	−17.38
Time in Risk Zone (s)	7.36	14.97	+103.40
Avg. Risk per Distance	1364.05	824.8	−39.53
Average Acceleration (m/s^2^)	11.39	0.63	−94.49
Peak-to-Mean Jerk Ratio (m/s^3^)	37.07	13.49	−63.61
Computation Time (s)	10.89	0.35	−96.82

**Table 6:** Relative impact of latency on path planning performance.

Category	GT-PF	GT-PF with Latency	Relative Difference (%)
Arrival Time (s)	34.36	34.42	+0.17
Travel Distance (m)	572.69	572.65	−0.01
Minimum Safety Distance (m)	4.04	3.72	−7.92
Time in Risk Zone (s)	14.97	15.08	+0.73
Avg. Risk per Distance	824.8	831.56	+0.82
Average Acceleration (m/s^2^)	0.63	0.69	+9.73
Peak-to-Mean Jerk Ratio (m/s^3^)	13.49	13.39	−0.74
Computation Time (s)	0.35	0.34	−1.42

## Data Availability

The datasets generated and analyzed in this study are part of an ongoing research project and are currently being used for further publications. Therefore, the data are not publicly available at this time. However, the raw data supporting the conclusions of this article will be made available by the authors upon reasonable request.

## Abbreviations

The following abbreviations are used in this manuscript:

RSU	Road Side Unit
RRT	Rapidly exploring Random Tree
MEC	Multi-access Edge Computing
PF	Potential Field
V2X	Vehicle-to-Everything
PRM	Probabilistic Roadmap Method
MPC	Model Predictive Control
iLQR	Iterative Linear Quadratic Regulator
DWA	Dynamic Window Approach
TTC	Time To Collision
SDM	Safety Distance Margin
